# Identification of amino acid variation in the prion protein associated with classical scrapie in Canadian dairy goats

**DOI:** 10.1186/s12917-016-0684-x

**Published:** 2016-03-22

**Authors:** Vythegi Srithayakumar, Gordon B. Mitchell, Bradley N. White

**Affiliations:** Natural Resources DNA Profiling and Forensics Centre, DNA Building, Trent University, 2140 East Bank Drive, Peterborough, ON Canada; National and OIE Reference Laboratory for Scrapie and CWD, Ottawa Laboratory Fallowfield, Canadian Food Inspection Agency, 3851 Fallowfield Road, Ottawa, ON Canada; Livestock Gentec, Department of Agricultural, Food and Nutritional Science, University of Alberta, Edmonton, AB T6G2P5 Canada

**Keywords:** Scrapie, Goat, Prion, Resistance, Susceptibility, Breeding, Variation

## Abstract

**Background:**

A clear association of amino acid variation in the prion protein gene (*PRNP*) with susceptibility and resistance to classical scrapie exists in sheep, but not in goats. In this study we examined DNA sequence variation in the *PRNP* of 149 animals from two scrapie-infected herds of Saanen dairy goats, and identified 6 non-synonymous variants in the coding region.

**Results:**

In the larger herd, all of the 54 scrapie-affected goats tested had at least one allele with the arginine (R) codon at position 211, with 52 being homozygous for that variant. No animal homozygous for the glutamine (Q) codon at 211 were affected and only two heterozygotes (R/Q) were affected. A weak association was found at position 146 and no significant associations were found with amino acid variation at the remaining four variant positions (142, 143, 222 and 240), however, the allelic variation was low. Similar patterns were observed in the second scrapie-affected herd.

**Conclusion:**

We also evaluated previous studies on goat herds affected with scrapie and this relationship of R susceptibility and Q resistance at 211 was present independent of the genotypes at the other positions including 222. The fact that glutamine at 211 provides a significant protective property to scrapie irrespective of the other positions could be important for breeding strategies aimed at improving herd resistance to scrapie, while maintaining important productivity traits.

## Background

Scrapie is a fatal neurodegenerative disease that affects sheep and goats. It is classified as a transmissible spongiform encephalopathy (TSE) belonging to a group of prion diseases, which includes Creutzfeldt-Jakob disease in humans, bovine spongiform encephalopathy (BSE) in cattle, and chronic wasting disease in cervids. Prion diseases not only have a serious impact on health and welfare, but control of these diseases impacts animal movement and trade. Prion diseases can occur sporadically as well as through heredity or infectious transmission routes [1] but pathogenesis is contingent on conversion of the normal host prion protein (PrP^C^) to a disease-associated form (PrP^Sc^). In sheep and goats, scrapie is characterized by the deposition of this abnormal, protease resistant prion protein (PrP^Sc^) in the central nervous system and peripheral tissues [2, 3]. The host prion gene (*PRNP*) encodes the prion protein (PrP^C^) and mutations within this gene have been associated with differential resistance and susceptibility to scrapie (as reviewed [4]).

Genetic resistance to scrapie is well established in sheep and three-codon *PRNP* genotypes have typically been utilized for risk assessment (as reviewed in [4]). The association of these three amino acid codons at positions 136, 154 and 171 produces 5 haplotypes (ARQ, VRQ, AHQ, ARR, ARH) and 15 genotypes [4, 5]. The ARR haplotype is associated with high resistance, whereas VRQ or ARQ are associated with higher susceptibility to scrapie [6, 7]. Sheep breeding programs selecting for the ARR genotype have been widely used for scrapie control and eradication programs in Europe and North America [8]. Patterns of associations to scrapie resistance and susceptibility based on the three codons used for sheep do not hold true in goats.

Although the natural occurrence of scrapie in goats appears generally lower than that in sheep, active surveillance reports suggest that the prevalence rates in goats may be underestimated in some countries [9]. There have been a number of studies that examined resistance and susceptibility to scrapie in goats from European countries (e.g., France, Italy, United Kingdom, Greece), where the goat populations are large and the incidence of scrapie has been high. These studies have characterized *PRNP* sequence variation in goats, and have reported a number of polymorphisms in goat populations worldwide [10–25]. In particular, thirty nine amino acid substitutions have been described, of which only six G127S [18], I142M [26], N146S/D [12], H154R [10, 12, 14, 19], R211Q [19], and Q222K [11, 14, 19] have been associated with resistance or susceptibility to scrapie in goats. At least 16 synonymous silent mutations have also been documented. To date, 39 out of the 256 codons have been shown to be polymorphic across goat breeds and generally 6 to 12 are described as polymorphic within a given goat breed. While some of the polymorphic sites are similar in sheep and goats, species-specific variation, like that observed for A_136_R_154_R_171_, are also present (as reviewed in [4]).

Despite the high amount of variation observed in the *PRNP* in various goat breeds, clear associations with scrapie susceptibility or resistance are limited. In the studies that have suggested associations with *PRNP* polymorphisms, the power of the analyses is often weak because of the relatively low frequencies of the putative resistant allele and the emphasis on identifying resistance alleles rather than susceptibility alleles. The unaffected animals represent a mixture of resistant and susceptible animals of varying exposure status, while the affected animals are more homogeneous in susceptibility traits. The absence of clearly identified variation, which is associated with scrapie in goats, has limited the implementation of breeding strategies to eliminate scrapie sensitive alleles in goat herds.

The objectives of this study were: (i) to identify the variation in *PRNP* genotypes in 2 herds of Saanen dairy goats with scrapie affected animals; (ii) to examine associations between PrP^C^ amino acid variation and scrapie disease in the largest herd and (iii) to validate the associations using the second scrapie affected herd together with published associations.

## Methods

### Scrapie affected herds

The first scrapie affected dairy goat herd was composed of 331 Saanen goats greater than 12 months of age. The herd was depopulated as part of routine disease control measures following the detection and confirmation of a scrapie-positive goat. Jugular blood was sampled in EDTA-vacutainer tubes for DNA extraction and the obex and retropharyngeal lymph nodes were removed for scrapie diagnostic testing. Initial testing of obex and lymph node tissues was conducted using a commercially available ELISA (Bio-Rad TeSeE^®^ ELISA, Bio-Rad Laboratories, Hercules, CA). Confirmatory testing was subsequently performed using immunohistochemistry with antibodies F89 and F99, and western immunoblot (Bio-Rad TeSeE^®^ western immunoblotting kit, Bio-Rad Laboratories) as previously described [27]. An animal was considered positive for scrapie if it was positive in at least one tissue by the confirmatory tests. A total of 66 animals were found positive for classical scrapie. *PRNP* was sequenced for 54 of the 66 scrapie-affected and 56 of the unaffected animals.

A second, smaller scrapie-affected herd that comprised 130 Saanen goats greater than 12 months of age was also identified and subjected to depopulation. Following the testing regime described above, 13 animals were identified as classical scrapie positive. *PRNP* was sequenced for 10 scrapie-affected and 29 unaffected animals.

#### PRNP sequencing and genotyping

Genomic DNA was extracted from EDTA-preserved whole blood using the MagNa Pure DNA Isolation Kit for blood (Roche Applied Sciences) as per manufacturer’s instructions, and was quantified using PicoGreen. The Goat *PRNP* reference sequence was obtained from Genbank (accession # X74758) and primers were designed to amplify the ORF of *PRNP* (PRNP2-F: AGCTGATGCCAGTGCTATGC and PRNP4R: GTGGCCTCCTTCCAGACTTG).

PCR buffer components were in the following concentrations: 1× PCR buffer (Invitrogen), 0.2 mM of each dNTP, 1.5 mM MgCl_2_, 0.3 μM of forward primer, 0.3 μM of reverse primer, 0.05 U/μL of Taq DNA polymerase (Invitrogen), 10 ng of DNA and ddH2O for a total volume of 20 μl. PCR conditions used for amplification were as follows: initial denaturation at 94 °C for 5 min; [denaturation at 94 °C for 30 s, annealing at 60 °C for 1 min, extension at 72 °C for 1 min 30 s] × 35 cycles; final extension at 72 °C for 15 min; and 4 °C hold. Two μl of the amplified product were subjected to agarose gel electrophoresis to confirm amplification. PCR products were purified using ExoSAP (New England BioLabs) following the manufacturer’s instructions. BigDye® Terminator v3.1 Cycle Sequencing Kit (Applied Biosystems) was used to sequence the fragments, using both the forward and reverse primers. Sequences were edited and aligned to Ovine and Caprine *PRNP* using MEGA™ 6.0 [28].

### Analysis of genotypes in affected and unaffected samples

Comparison of the genotype frequencies of affected and unaffected groups were carried out using logistic regression models fitted to scrapie status of goats in the affected groups. When there were no positive scrapie cases, Fisher’s exact test was used.

#### Relative susceptibility of genotypes to scrapie

Herd 1 consisted of 331 animals more than 12 months of age and 66 tested scrapie positive; the remaining 265 animals, consisted of animals that tested scrapie negative on ELISA. The scrapie negative animals may have been exposed to the disease and not succumbed or simply may not have been exposed to the disease. In order to estimate the relative susceptibility of different genotypes to scrapie, the frequencies of alleles and genotypes in the pre-exposed herd one were estimated. We assumed the genotypes of the 56 unaffected samples that were genotyped were representative of the 209 unaffected animals that were not genotyped and the genotypes of the 54 affected animals were representative of the12 affected animals that were not genotyped. The percentages of each genotype in the pre-exposed herd that became scrapie positive were compared.

## Results

### PRNP variation and allele frequencies in 2 scrapie affected Ontario Saanen herds

**Fig. 1:**
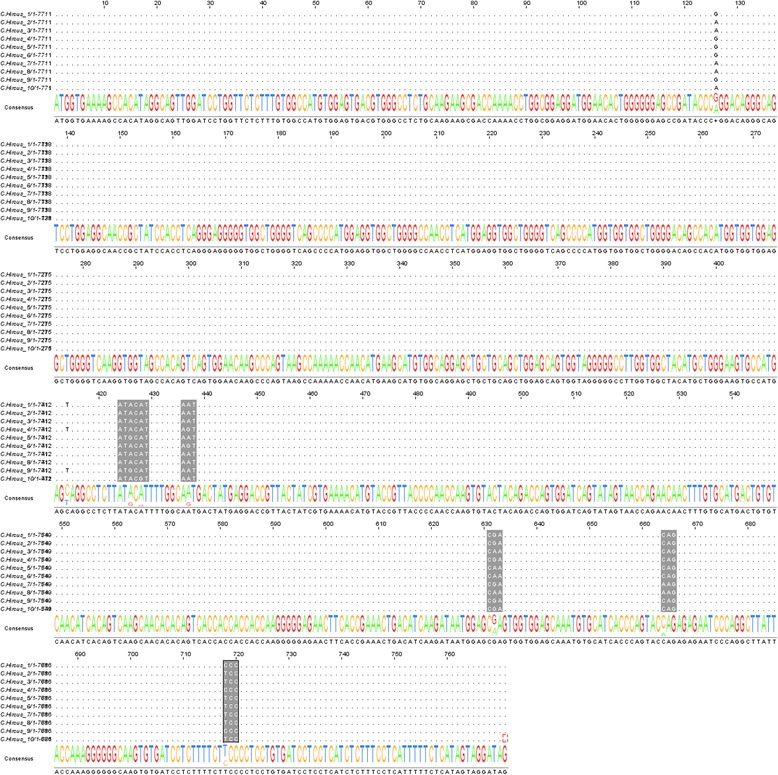
Nucleotide sequences of the eight variable sites for the ten haplotypes observed in the Ontario Saanen herds. C. hircus *PRNP* (genbank accession # X74758.1) was used as the reference. Synonymous substitutions are denoted in lower case

The open reading frames of the *PRNP* [5] were sequenced in 54/66 of the affected animals and 56/265 of the unaffected animals. A total of eight variable sites were found, with six non-synonymous substitutions (codon positions: 142, 143, 146, 211, 222 and 240) and two synonymous substitutions (Fig. 1). Amino acids are designated by the single letter codes that give six letter alleles or haplotypes. Initially allele haplotypes were assigned based on homozygous animals; heterozygous animals were genotyped based on the confirmed alleles in the homozygotes. This left haplotype uncertainty in three of the ten alleles that were in low frequency, (Table 1) and the genotypes with those alleles (Table 2). The haplotype frequencies for each of the two herds show a high prevalence of four alleles (Table 1).

**Table 1:** Allele frequencies for *PRNP* in herds 1 and 2

Amino acid position							Haplotype frequency	
	142	143	146	211	222	240	Herd 1	Herd 2
1	I	H	N	R	Q	P	0.459	0.526
2	–	–	–	–	–	S	0.305	0.231
3	–	–	–	Q	–	S	0.159	0.090
4	–	–	S	–	–	–	0.032	0.090
5^¥^	M	–	–	Q	–	S	0.005	0.000
6^¥^	–	–	S	Q	–	S	0.005	0.000
7^¥^	–	–	–	Q	K	S	0.005	0.000
8	–	–	–	–	K	S	0.014	0.000
9	M	–	–	–	–	–	0.014	0.064
10	–	R	–	–	–	S	0.005	0.000

¥ unconfirmed haplotypes

**Table 2:** Comparison of genotypes among the complete herd, affected and unaffected animals in herd 1. Logistic regression was used to compare affected and unaffected animals

*PRNP* genotype	Pre-exposure^a^	Scrapie positive sample	Scrapie negative sample	OR (95 % CI)	*P*-value
IHNRQP/IHNRQP	53	16	7	2.947 (1.089–7.973)	0.033
IHNRQS/IHNRQP	63	28	6	8.974 (3.261–24.699)	<0.005
IHNRQS/IHNRQS	13	7	1	8.191 (0.949–70.704)	0.055
MHNRQP/IHNRQS	14	0	3	ND^ǂ^	–
MHNQQS/IHNRQP	5	0	1	ND	–
IHNRQP/IRNRQS	1	1	0	ND	–
IHSRQP/IHNRQP	9	0	2	ND	–
IHSRQP/IHNRQS	14	0	3	ND	–
IHSRQP/IHSRQP	5	0	1	ND	–
ihsqqs/IHNRQP	5	0	1	ND	–
IHNQQS/IHNRQP	67	1	14	0.057 (0.007–0.459)	0.007
IHNQQS/IHNRQS	44	1	9	0.098 (0.012–0.827)	0.033
IHNQQS/IHNQQS	24	0	5	ND	–
IHNRKS/IHNRQP	5	0	1	ND	–
IHNRKS/IHNRQS	5	0	1	ND	–
IHNRKS/IHNQQS	5	0	1	ND	–
Total	331	54	56	–	–

^a^number of genotypes pre-exposure was estimated as described in the methods

^ǂ^ OR could not be determined (ND) for genotypes without any scrapie positive cases

### Association of variants with scrapie susceptibility

We compared the genotypes of affected and unaffected animals in order to assess the association with scrapie susceptibility using logistic regression (Table 2). The genotypes IHNRQP/IHNRQP and IHNRQS/IHNRQP, were seen in significantly higher frequency in scrapie positive animals than scrapie negative animals. Conversely, IHNQQS/IHNRQP and IHNQQS/IHNRQS were present predominantly in scrapie negative animals. Next, we examined the relative susceptibility of the different genotypes to scrapie.

We focused on the affected animals, as the unaffected sample represents a mixture of potentially resistant animals as well as unexposed or less exposed animals (Table 2). The homozygous genotype IHNRQP/IHNRQP was estimated to be present in 53 individuals in the pre-exposed herd and 16 were found in the scrapie positive sample, while the heterozygote IHNQQS/IHNRQP was estimated to be present in 67 individuals prior to exposure and only one was found in the scrapie positive samples. In order to identify the amino acids conferring scrapie susceptibility we analysed the six variable positions individually.

The genotype frequencies of the affected animals were compared to the expected frequencies based on the pre-exposed herd and the null hypothesis that the affected animals represent a random sample of genotypes in the herd (Table 3). A significant difference was found at position 211, and a weaker significance at 146. Arginine (R) at 211 was clearly associated with susceptibility while glutamine (Q) was associated with resistance in the RQ_211_ heterozygote. There were fewer affected animals in herd 2 but all were RR_211_, consistent with R being a susceptibility allele (Table 3).

**Table 3:** Comparison of observed and expected numbers of scrapie positive samples for each amino acid variant. Allele frequencies of the pre-exposed herd are indicated in parentheses. *P*-values of <0.05 are indicated in bold. Chi-square analysis was used for herd 1, and Fisher’s exact test was used for herd 2 as the sample size was low

		Numbers in Scrapie positive samples			
		Herd #1		Herd #2	
Codon	Genotype	Observed	Expected^≠^	Observed	Expected^≠^
142	II (0.915)	54	51	10	8
	IM (0.085)	0	3	0	2
	MM (0.000)	0	0	0	0
143	HH (0.997)	53	54	10	10
	HR (0.000)	0	0	0	0
	RR (0.003)	1	0	0	0
146	NN (0.876)	54	49	10	8
	NS (0.105)	0	5	0	2
	SS (0.019)	0	1	0	0
211	RR (0.615)	52	30	10	8
	RQ (0.333)	2	20	0	2
	QQ (0.051)	0	4	0	0
222	QQ (0.969)	54	52	10	10
	QK (0.021)	0	2	0	0
	KK (0.010)	0	1	0	0
240	PP (0.295)	16	12	3	5
	PS (0.465)	30	28	7	3
	SS (0.239)	8	15	0	2

^≠^Allele frequencies for the expected numbers were calculated using the frequencies of the pre-exposed herd

When we examined the genotype estimates of the pre-exposed herd at position 211, the relative risk of developing the disease for the genotype RR_211_ and RQ_211_ was clear, with 64 of 182 RR_211_ being affected while only two of 125 RQ_211_ were affected. These data highlight the protective effect of Q in the heterozygote making it approximately 22 × more resistant than RR (Table 4).

**Table 4:** Comparison of the genotypes at position 211 to assess the relative scrapie risk

Genotype	Pre-exposed herd^ǂ^	Scrapie positive^ǂ^	Scrapie negative^ǂ^	Relative risk
RR	182	64	118	22
RQ	125	2	123	1
QQ	24	0	24	0

^ǂ^Total number of animals in each group, were calculated using the frequencies of the pre-exposed herd

The results in Tables 3 and 4 show a clear association between R_211_ and scrapie susceptibility. In the literature there are reported patterns of resistance for M_142_, S_146_, R_211_ and K_222_ [11, 14, 19, 21, 29, 30], but the power of the analyses for these codons are often weak because of low allele frequencies of the proposed resistant alleles (Table 5). We examined the 544 scrapie affected samples described in the literature, and this showed most are homozygous for the proposed susceptibility alleles at positions 142, 146, 211 and 222. This is because the resistant alleles are in very low frequency in most herds in Europe and the US [11, 13, 14, 19, 21, 29, 30] and emphasises the importance of establishing the allele frequencies in the herds when associating resistance and susceptibility with genotypes. As with the data presented here, the heterozygote RQ_211_ is found significantly less frequently than homozygous RR_211_, and the QQ_211_ genotype was not seen in any affected animal.

**Table 5:** Comparison of genotypes affected with scrapie from the literature. A total of 544 scrapie cases from various breeds were assessed

Number of affected	142			146			211			222			References
	II	IM	MM	NN	NS	SS	RR	RQ	QQ	QQ	QK	KK	
30	83	1	0	-	-	-	83	7	0	87	3	0	Barillet et al. 2009
259	238	21	0	-	-	-	246	13	0	256	3	0	Corbiere et al. 2013
39	-	-	-	-	-	-	-	-	-	39	0	0	Vaccari et al. 2006
27	-	-	-	-	-	-	27	0	0	27	0	0	Bouzalas et al. 2010
25	-	-	-	-	-	-	-	-	-	25	0	0	Acutis et al. 2006
104	103	1	0	104	0	0	104	0	0	99	5	0	Fragkiadaki et al. 2011
*Total:* *544*	*424*	*23*	*0*	*104*	*0*	*0*	*460*	*20*	*0*	*533*	*11*	*0*	-

## Discussion

Scrapie poses a threat to the Canadian goat industry as herds on farms that test positive are entirely eradicated, and its presence limits the international export of goat genetics. Control of scrapie in sheep and goats requires knowledge of the *PRNP* genotypes which influence disease susceptibility. In sheep there is a clear association of amino acid variation at three positions in *PRNP* to susceptibility and resistance to scrapie. There are weaker associations with four positions in goats and this seems to be caused by low frequencies of variants, which in turn impact the power of the analyses. Analyses have also focused on identifying resistant alleles in unaffected animals that represent a mixture of resistant and unexposed animals. In this study, we had samples from a large herd of Saanen dairy goats with 66 affected individuals and a smaller herd with 13 affected animals.

The previous studies on goat scrapie have identified a number of variants in the different breeds and herds, which are associated with a lower incidence of scrapie [10–12, 14, 19, 26]. These include M_142_, S_146_, Q_211_ and K_222_, which were present in the two herds we examined; but apart from Q_211_, the frequencies of the other variants were low. However, these variants were only found in the unaffected animals in both herds.

### PRNP variation in Ontario Saanens

Thirty-seven amino acid substitutions have been previously described in the open reading frame of *PRNP* in goats around the world. The six polymorphisms found in the Saanens from Ontario have been previously described [11, 13, 14, 19, 26]. It is usual to observe only a subset of the global goat *PRNP* polymorphisms in a given breed or herd. Previous studies have found polymorphic positions ranging from 5 to 14 [11, 13, 18–20]. Saanens often have six polymorphic codons, with the specific position differing among different herds in different countries. Three of the six (142, 211, and 240) were found in Italian Saanens; four (142, 211, 222 and 240) were found in the French and Spanish Saanens. The remaining two polymorphic codons 143 and 146 that were seen in low allele frequencies (0.34 and 5 %, respectively), have been reported in Saanens crossbred with Hellenic and Damascus breeds, respectively [10, 12, 24]. As the Saanen breed originated from Switzerland, these similarities and differences are the result of founder effects combined with cross breeding with local goat breeds.

Of the ten haplotypes found in this study, three (IHNRQP, IHNRQS and IHNQQS) were present in 90 % of the sampled animals. The two major *PRNP* haplotypes (IHNRQP and IHNRQS) differed from one another by the presence of proline (P) or serine (S) at position 240. In both of the herds investigated, P_240_ was slightly higher in frequency (55 %) compared to S_240_ (45 %). This pattern has been noted in French Saanens [19, 21], Italian [11, 14], Spanish [20], British [18]; North American [13] and Greek goats [10, 23, 29, 30]. When we estimated the haplotype and genotype frequencies for all the animals in both herds, comparable trends were observed for the two main haplotypes (IHNRQP and IHNRQS); however, for the other haplotypes present in low frequencies, significant differences were present between the two herds. Low haplotypic frequencies are usually encountered when examining *PRNP* variation in goat herds [10, 11, 14, 19–21, 23].

### PRNP variants associated with scrapie susceptibility

When we compared the genotypes of affected and unaffected animals, we found two genotypes to be associated with scrapie susceptibility and two to be associated with resistance. Specifically, we compared the estimated allele and genotype frequencies of the herd prior to exposure with those of affected animals. Of the six variable positions, two (146 and 211) had genotype frequencies significantly different in the affected samples than those expected from the pre-exposed frequencies. There was weak significance for position 146 and similar results have been found in other studies, where the homozygous NN_146_ genotype has been associated with scrapie susceptibility in Cypriot goats [16, 24]. The homozygous RR_211_ genotype was strongly associated with scrapie susceptibility, with the heterozygous RQ_211_ genotype conferring a degree of protection to the disease.

### Effect of position 211 on scrapie susceptibility and resistance

All the affected animals from herd 1 had at least one allele with arginine (R) at position 211. Specifically, of the 54 affected samples tested, 52 were RR_211_, whereas, the remaining 2 were RQ_211_. In the unaffected group, RR_211_ was seen in 25 of the 56 samples and RQ_211_ and QQ_211_ were seen in 26 and 5 animals, respectively. A similar result was seen in the second herd where all the affected samples were RR_211_. These findings indicate that glutamine (Q) at position 211 confers protection to scrapie. The frequency of Q is usually low and therefore the homozygous QQ_211_ is seen in very low frequency in most herds, however it has not been seen any of the affected animals reported in the literature (Table 5).

Significant differences between the frequencies of the RQ_211_ in the unaffected samples compared to the affected samples shows heterozygous goats have a significantly lower susceptibility to scrapie than RR_211_ individuals. A comparison of the estimated genotypes of the herd prior to scrapie exposure to the affected animals shows 35 % of the homozygous RR_211_ individuals developed the disease, whereas only 1.6 % of the heterozygous RQ_211_ individuals succumbed. Similar patterns have been reported in the literature. In French goat populations, the risk of succumbing to scrapie for RR_211_ and RQ_211_ to be 30–39 % and 4.4–14 %, respectively [19, 21]. These results highlight the protection provided by the glutamine (Q) at position 211 and low prevalence of the disease in the heterozygous animals.

Although additional studies are required, it is interesting to compare the 211 variant position in goats to position 171, of the A_136_R_154_Q_171_ allele in sheep. In sheep, homozygous ARR is considered resistant and homozygous ARQ is susceptible; whereas ARR/ARQ heterozygous sheep are relatively resistant to scrapie [7, 31]. Similar results were found in this study where homozygous QQ_211_ and RR_211_ were found resistant and highly susceptible respectively, while there was a very low prevalence of scrapie in RQ_211_ heterozygous animals. Although, scrapie has not been seen in Canadian ARR/ARQ sheep to date, it has been detected in Europe sheep [31–33]. Statistical models have estimated that genotypes with ARR haplotypes were 1000× more resistant to the disease compared to homozygous ARQ [33, 34]. In this study we estimated RQ_211_ were 22 × more resistant than homozygous RR_211_, suggesting that RQ_171_ in sheep has a stronger influence on resistance than RQ_211_ in goats. Similar to homozygous ARR sheep, the QQ_211_ genotype in goats is seen in relatively low frequency [31–33].

Although the mechanism for the conversion of PrP^C^ to the pathogenic form (PrP^Sc^) and its pathological effects are poorly understood, specific mutations in sheep *PRNP* associated with resistance are shown to destabilize the PrP^C^ protein [35]. Direct correlation of scrapie susceptibility and mutation induced changes are unlikely as the structural consequence of scrapie associated variants are local. As such, variations in scrapie susceptibility are likely caused by altering protein stability. Destabilizing the structure of PrP^C^ may increase protease sensitivity and slow amyloidogenesis, thereby preventing PrP^C^ from being converted to the pathogenic form. This is thought to account for the change from scrapie susceptibility to resistance following a single amino acid substitution (ARQ_171_ to ARR_171_) in sheep [36], and perhaps also the inhibition of cell-free conversion following the substitution of R to Q at codon 211 in recombinant goat PrP [37].

### Position 211 and developing scrapie resistant dairy goat herds in Ontario

An association of other variants (M_142_, S_146_ and K_222_) with protection towards scrapie was not tested in this study due to low allele frequencies of the potentially resistant allele. QQ_211_ provided protection regardless of the amino acids at the other positions such as 222. There is a suggestion from challenge studies that K_222_ may confer more resistance than Q_211_ [22]. In preliminary studies on Ontario dairy goat herds, the frequency of K_222_ has been found to be very low or absent, making elimination of Q_222_ very difficult. The extent to which elimination of R_211_ could result in scrapie resistant herds is of great interest and additional studies on surveying genotype frequencies in Ontario dairy herds are underway.

## Conclusions

The data presented, highlights the association of arginine (R) at position 211 with susceptibility to scrapie and the protective effect of Q, irrespective of the amino acids at the other positions. To date there are no programs in place in Canada to select for resistance towards scrapie in goats. A major barrier may be the low frequency of scrapie resistant animals in Canadian goat herds. As the male to female ratio in breeding programs is often 1:20, the genotype of the bucks has a strong impact on the determination of the overall genetic profile of the herd. The most efficient strategy to select against high risk animals, at the same time minimizing the loss of production traits, would be to only utilize more resistant bucks or semen. This would be a particularly useful approach for goat producers associated with a farm previously infected with scrapie, and goat producers providing large numbers of breeding ewes to other producers.

In sheep, breeding for scrapie resistant with ARR/ARR rams has been a successful strategy for the prevention of scrapie outbreaks [38]. Furthermore, increased frequencies of the resistance associated ARR allele, as a result of selective breeding, is assumed to have a population effect by reducing the scrapie infection risk even for animals of susceptible genotypes [8, 39]. As the protection provided by R_171_ in heterozygous sheep seems to be several folds higher than the protection provided by Q_211_ in heterozygous goats, selection for homozygous QQ_211_ goats should be favoured. Breeding for resistance in goats by recruiting bucks with scrapie-resistant genetics, should increase the frequency of resistant alleles in the herd, with a resultant reduction in classical scrapie susceptibility.
